# Neutrophils Are Central to Antibody-Mediated Protection against Genital Chlamydia

**DOI:** 10.1128/IAI.00409-17

**Published:** 2017-09-20

**Authors:** Elizabeth K. Naglak, Sandra G. Morrison, Richard P. Morrison

**Affiliations:** Department of Microbiology and Immunology, University of Arkansas for Medical Sciences, Little Rock, Arkansas, USA; Washington State University

**Keywords:** chlamydia, genital tract immunity, immunity, sexually transmitted diseases

## Abstract

Determining the effector populations involved in humoral protection against genital chlamydia infection is crucial to development of an effective chlamydial vaccine. Antibody has been implicated in protection studies in multiple animal models, and we previously showed that the passive transfer of immune serum alone does not confer immunity in the mouse. Using the Chlamydia muridarum model of genital infection, we demonstrate a protective role for both Chlamydia-specific immunoglobulin G (IgG) and polymorphonuclear neutrophils and show the importance of an antibody/effector cell interaction in mediating humoral immunity. While neutrophils were found to contribute significantly to antibody-mediated protection *in vivo*, natural killer (NK) cells were dispensable for protective immunity. Furthermore, gamma interferon (IFN-γ)-stimulated primary peritoneal neutrophils (PPNs) killed chlamydiae *in vitro* in an antibody-dependent manner. The results from this study support the view that an IFN-γ-activated effector cell population cooperates with antibody to protect against genital chlamydia and establish neutrophils as a key effector cell in this response.

## INTRODUCTION

Chlamydia trachomatis, the most common sexually transmitted bacterial infection worldwide, is responsible for more than 90 to 100 million infections annually, and health care costs exceed $500 million/year in the United States alone (1–3). Particularly alarming are the high rates of asymptomatic cases, with more than 50% of men and up to 95% of women reporting no noticeable symptoms (4). Genital infection results in a variety of pathological consequences in women, which range from mild cervicitis to pelvic inflammatory disease, ectopic pregnancy, and infertility (5–7). However, because of the asymptomatic nature of this pathogen and the serious pathological consequences that can result from untreated infection, renewed efforts have been placed on vaccine development. To develop a protective vaccine, it is important to understand the natural immunity that develops following genital infection resolution in animal models. The highly related Chlamydia muridarum murine genital infection model has been widely used because it recapitulates in many aspects both the pathology and ascension of genital infection seen in women.

Previous studies using the C. muridarum murine genital infection model have shown convincingly that both cellular and humoral arms of the adaptive immune response contribute to protective immunity (8–10). By analyzing the immunity that develops following primary infection, it has been shown that protection against reinfection is multifactorial, with CD4^+^ T cells and antibody playing predominant roles (9, 11–13). While CD4^+^ T cells themselves are protective, studies have concluded that Chlamydia-specific antibody is unable to effectively directly neutralize C. muridarum *in vivo*, suggesting that antibody interaction with an effector cell population(s) is required for bacterial killing (9, 11, 13).

Antibody interacts with immune effector populations in several different ways to mediate protection against pathogens, though each requires the Fc region to bind a cognate Fc receptor (FcR) on an effector cell. Antibody-dependent cellular cytotoxicity (ADCC) is one common mechanism of defense, where nonself antigen is presented on the surface of infected cells and opsonized by antigen-specific antibody. Typically natural killer (NK) cells then recognize and bind the unbound Fc portion of the antibody, triggering a degranulation cascade that results in target cell lysis. Occasionally, ADCC has also been found to occur via macrophages, neutrophils, and eosinophils (14). Both ADCC and NK cells have been implicated in protective immunity to genital chlamydia (15, 16).

In addition to ADCC, antibody also participates in pathogen clearance via pathogen/antibody complex internalization and subsequent pathogen killing by phagocytes. Pathogen-specific antibodies first bind their cognate antigen, and then immune phagocytic cells (neutrophils, macrophages, and also dendritic cells) bind to the free Fc region of the antibody/antigen complex, leading to phagocytosis of the entire complex. The pathogen will then be shuttled along the endocytic/lysosomal pathway, ultimately leading to its degradation. Support for this mechanism of protection includes antibody-mediated protection's requirement for gamma interferon (IFN-γ) (17), which upregulates expression of FcRs and enhances microbial killing in phagocytes (18, 19).

In the current study, we established that the immunoglobulin G (IgG) isotype was specifically responsible for this protection. Additionally, we showed that NK cells (and their degranulation) were dispensable for humoral immunity. In contrast, our data indicated a significant role for neutrophils, in conjunction with antibody, in mediating protection, thus supporting the hypothesis that antibody/effector cell interactions function in antibody-mediated immunity to genital chlamydia reinfection.

## RESULTS

### IgG confers antibody-mediated immunity.

**FIG 1:**
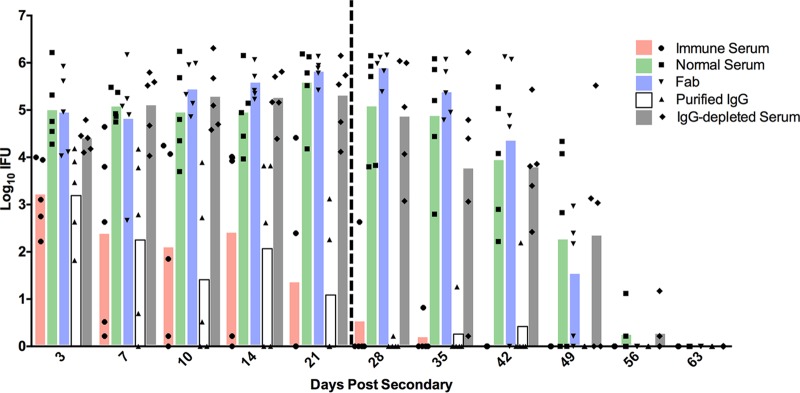
Antibody-mediated protection is conferred by IgG. Prior to and throughout secondary infection, immune serum (or a fraction of it) or normal serum was passively transferred to CD4-depleted antibody-deficient mice (*n* = 5). Following infection, IFU were quantitated from cervicovaginal swabs collected at various time points after secondary infection. The vertical dashed line at day 23 represents the final anti-CD4 injection. For clarity, statistical significance is listed here rather than on the figure: for normal serum versus immune serum, *P* < 0.01 for days 7, 10, and 14, *P* < 0.0001 for days 21 through 42, and *P* < 0.05 for day 49; for normal serum versus purified IgG, *P* < 0.01 for days 7 and 14, *P* < 0.0001 for days 10 and 21 through 42, and *P* < 0.05 for day 49. There were no statistical differences between normal serum and Fab serum, normal serum and IgG-depleted serum, or immune serum and purified IgG at any time point.

**FIG 2:**
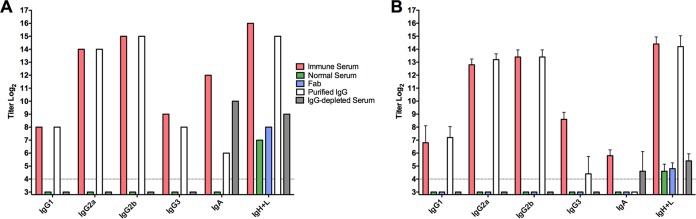
Class- and subclass-specific serum antichlamydial antibody titers prior to transfer and 16 days after passive transfer into CD4-depleted antibody-deficient mice. Anti-Chlamydia antibody titers were measure by ELISA using formalin-fixed EBs as the antigen. (A) Anti-Chlamydia titers of normal serum, immune serum, immune IgG, IgG-depleted immune serum, and immune Fab prior to passive transfer. (B) Anti-Chlamydia titers of serum collected 16 days after secondary infection (day 82) from CD4-depleted antibody-deficient mice that had received passively transferred serum or fractions thereof. Titers from individual mouse sera were determined, and data are presented as means ± standard deviations (SD) for 5 mice/group.

Immune (convalescent) serum, collected following the resolution of primary chlamydia genital infection, protects against reinfection (13). Those studies also demonstrated that the protection conferred by the immune serum was not due to the direct neutralization of the chlamydiae but likely was due to an antibody/effector cell interaction. To confirm that the protective efficacy of the immune serum was indeed IgG mediated and to investigate the role of the immunoglobulin Fc region in the protective antibody response, serum was fractionated and tested for protective efficacy using the well-defined murine model of chlamydia reinfection. Prior to secondary infection, normal mouse serum, immune serum, protein A-purified immune IgG, IgG-depleted serum, or Fab fragments were passively transferred to antibody-deficient (μMT) mice depleted of CD4^+^ T cells. Antibody-deficient mice depleted of CD4^+^ T cells are incapable of resolving secondary infection naturally because they are deficient in both protective arms (humoral and cellular) of antichlamydial immunity (9, 11–13). Using this infection model, any protection observed must be afforded to the mice by the passively transferred serum. Immune serum and purified immune IgG significantly protected the mice against secondary infection (Fig. 1). However, normal serum, immune serum depleted of IgG (protein A flowthrough), or Fab fragments prepared from immune IgG were unable to protect against infection, as these mice displayed a heightened, persisting infection that did not begin to resolve until after CD4^+^ T cell depletion had ended. Consistent with the lack of direct neutralizing ability of antibody *in vivo* (13), these data indicate that IgG, rather than IgM or IgA (protein A flowthrough fraction), conferred protective immunity and that the Fc region of Chlamydia-specific Ig was required for antibody-mediated protection. A Chlamydia-specific enzyme-linked immunosorbent assay (ELISA) was used confirm equivalent antibody titers prior to passive transfer (Fig. 2A) and at 16 days after secondary infection (Fig. 2B). Taken together, these data directly show that IgG is a protective component of the antichlamydial humoral response and that protection is dependent on an intact antibody.

### Antibody mediates chlamydial clearance independently of NK cells.

Cleavage of the Fc region of antichlamydial antibodies resulted in complete ablation of the antibody's protective capacity, suggesting that direct interaction with an effector population(s) is required for antibody-mediated immunity. To identify the effector cells that might be involved in antibody-mediated protection, we first considered the immune cell populations expressing FcRs, which are required for antibody/effector cell interactions. *In vitro* studies have suggested a role for ADCC in the killing of Chlamydia-infected epithelial cells (16), leading us to question whether NK cells (classical mediators of ADCC) play a role in antibody-mediated immunity.

**FIG 3:**
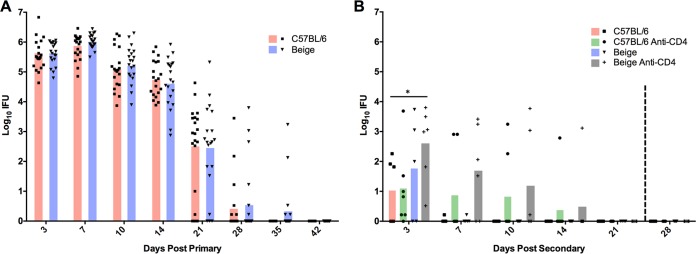
Primary and secondary C. muridarum infection in CD4-depleted and nondepleted C57BL/6 and beige mice. (A) Primary infection course in C57BL/6 (*n* = 20) and beige (*n* = 20) mice. Prior to secondary rechallenge, mice from each strain (from panel A) were depleted of CD4^+^ T cells. At 66 days after primary infection, all mice were rechallenged intravaginally with C. muridarum. (B) Secondary infection course in CD4-depleted (*n* = 6) and nondepleted (*n* = 5) C57BL/6 and beige mice. The vertical dashed line at day 27 represents the final anti-CD4 injection. There were no statistical differences between C57BL/6 and beige mice during primary infection. *, *P* < 0.05.

To determine whether NK cell-mediated ADCC contributed to antibody-mediated protection against genital chlamydia rechallenge, we utilized two models of NK cell deficiency: beige mice, which display a severe degranulation defect, resulting in a greatly dampened ability of immune granulocytes (including NK cells) to protect against a variety of pathogens, and the *in vivo* depletion of NK cells using specific antibody. The resolution of primary chlamydial genital infection of beige mice was indistinguishable by bacterial shedding and infection duration from that of C57BL/6 mice, suggesting that NK cells are not required for the resolution of primary infection (Fig. 3A). To test the protective efficacy of the Chlamydia-specific antibody response in beige mice following rechallenge, CD4^+^ T cells were depleted from mice of each strain prior to secondary infection. Fully immunocompetent C57BL/6 and anti-CD4-treated C57BL/6 mice were markedly protected against reinfection (9, 11, 13), exhibiting a significant decrease in both bacterial shedding and duration of infection compared to primary infection (Fig. 3B). Similarly, beige mice were also protected against reinfection, both with and without CD4^+^ T cell depletion. Those data support the view that immune cell degranulation (and therefore ADCC) is not a major mechanism of antibody-mediated protection.

**FIG 4:**
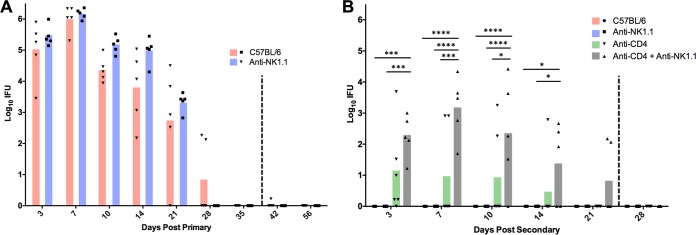
Effect of NK cell depletion on primary and secondary C. muridarum infection in CD4-depleted and nondepleted C57BL/6 mice. (A) Primary infection course in NK cell-depleted (anti-NK1.1) and nondepleted C57BL/6 mice. The vertical dashed line at day 38 represents the final anti-CD4 injection. (B) Secondary infection course in nondepleted C57BL/6 mice or in mice depleted of NK cells or NK cells and CD4^+^ T cells. The vertical dashed line at day 26 represents the final anti-CD4 and anti-NK1.1 injections. Experiments with CD4-depleted C57BL/6 mice from Fig. 3 ran concurrently with NK cell studies for Fig. 4 and thus are displayed again (green bars) for statistical comparison. There were no statistical differences between C57BL/6 and anti-NK1.1 mice during primary infection. *, *P* < 0.05; ***, *P* < 0.001; ****, *P* < 0.0001 (*n* = 5 mice for all experimental groups).

**FIG 5:**
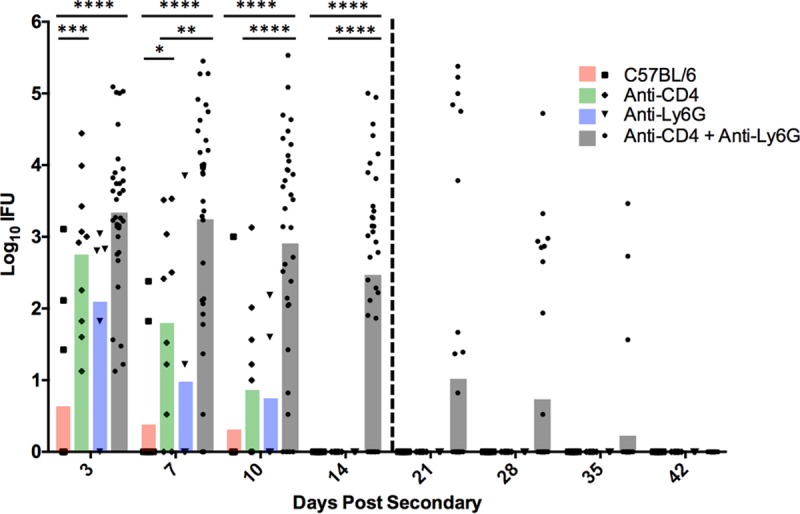
Effect of neutrophil depletion on bacterial clearance during secondary C. muridarum infection. At 66 days after primary infection, C57BL/6 mice were rechallenged. Prior to this reinfection, mice were left untreated (*n* = 10) or treated with anti-CD4 (*n* = 10), anti-Ly6G (*n* = 5), or anti-CD4 and anti-Ly6G (*n* = 19). IFU were quantified from cervicovaginal swabs collected throughout secondary infection. The vertical dashed line represents the last dose of anti-CD4 and anti-Ly6G at days 20 and 21, respectively. Data are presented as mean IFU from two independent experiments. *, *P* < 0.05; **, *P* < 0.01; ***, *P* < 0.001; ****, *P* < 0.0001.

To further assess the role of NK cells in the resolution of primary infection and in antibody-mediated immunity to rechallenge, C57BL/6 mice were depleted of NK cells using anti-NK1.1 prior to either primary or secondary challenge. NK-depleted mice exhibited primary infections comparable to those of nondepleted mice (Fig. 4A). During secondary infection, NK cell depletion alone had no effect on protective immunity, and CD4^+^ T cell depletion only minimally affected protective immunity. Although anti-CD4 + anti-NK1.1-treated mice were susceptible to reinfection, chlamydia shedding was less robust and the duration of infection was shorter than in primary infection of fully immunocompetent mice (Fig. 4B). Thus, considerable protective immunity remained following the depletion of both CD4^+^ T cells and NK cells and was not unlike what we have observed previously with anti-CD4 depletion (Fig. 5) (9, 11, 13). Notably, combined NK cell/CD4^+^ T cell depletion did not result in a prolonged persisting reinfection, such as that observed in CD4-depleted antibody-deficient mice, implying that NK cells are minimally involved and ultimately dispensable for antibody-mediated immunity to Chlamydia reinfection.

### Neutrophils contribute to antibody-mediated immunity.

In addition to NK cells, other immune effectors present in the genital tract during chlamydial infection express FcRs for antibody interaction (17). Previous data indicate a key role for IFN-γ activation in antibody-mediated immunity (17); thus, we chose to investigate a role for neutrophils, a predominant cell population responding to genital chlamydia infection and an IFN-γ-responsive effector population, in antibody-mediated chlamydial clearance. After primary infection but prior to secondary rechallenge, C57BL/6 mice were depleted of neutrophils (alone or in combination with CD4^+^ T cells) via anti-Ly6G administration. While neutrophil depletion alone led to a slight increase in bacterial burden, depletion of both CD4^+^ T cells and neutrophils resulted in significantly more bacterial shedding and persisting infection (Fig. 5), thus supporting the premise that neutrophils contribute importantly to antibody-mediated antichlamydial immunity.

### Chlamydia-specific antibody responses in neutrophil-depleted mice.

**FIG 6:**
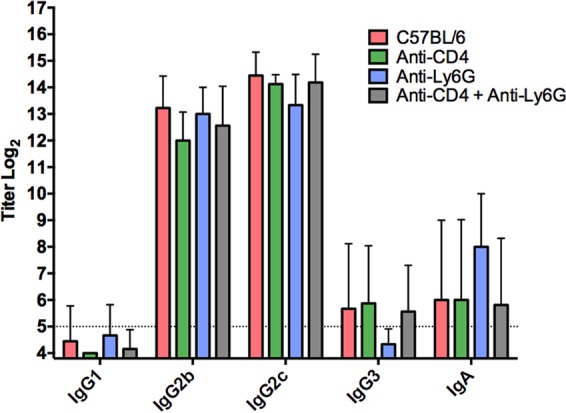
Class- and subclass-specific serum antichlamydial antibody titers of neutrophil- and CD4^+^ T cell-depleted mice during secondary infection. Serum was collected 24 days after secondary infection (90 days after primary infection) from anti-CD4 (*n* = 8)-, anti-Ly6G (*n* = 4)-, and anti-CD4/anti-Ly6G (*n* = 18)-treated mice, as well as from wild-type (untreated) C57BL/6 mice (*n* = 9). Chlamydia-specific ELISAs were performed using whole formalin-fixed EBs as the antigen. Titers from individual mice were determined, and data are presented as means ± SD.

To confirm that the results showing the involvement of neutrophils in antibody-mediated chlamydial immunity were not due to an altered antibody response in mice depleted of neutrophils and CD4^+^ T cells, serum was collected and analyzed by ELISA for Chlamydia-specific antibody. All class and subclass Chlamydia-specific titers were comparable between the groups of mice (Fig. 6), suggesting that a change in antibody production was not the cause for the diminished humoral protection displayed by neutrophil-depleted mice.

### Anti-Ly6G effectively depletes neutrophils at early time points postinfection.

**FIG 7:**
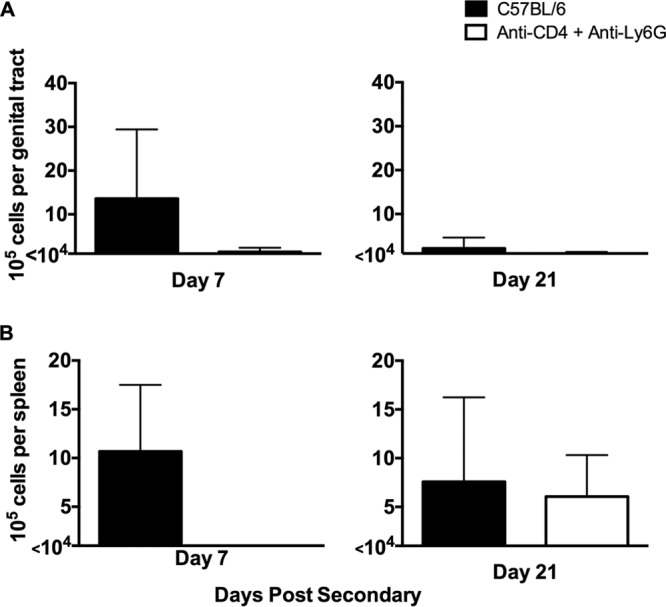
Effective depletion of neutrophils in genital tract tissue and spleens. Whole genital tracts (A) and spleens (B) were collected from nondepleted and CD4/Ly6G-depleted mice at days 7 (early) and 21 (late) after secondary infection. Viable neutrophils were isolated and stained with antibodies for flow cytometry. Data represent the mean viable number of positive-staining cells per genital tract (A) or spleen (B) ± SD from 5 individual mice per time point.

Although intraperitoneal (i.p.) administration of anti-Ly6G is known for its ability to deplete neutrophils (20), we wanted to ensure effective elimination of this population for the duration of secondary infection. Whole genital tracts and spleens were harvested from wild-type C57BL/6 mice and C57BL/6 mice treated with anti-CD4 and anti-Ly6G at days 7 (early) and 21 (late) after secondary infection. Mice treated with anti-CD4 are markedly depleted (9, 11, 17, 21), and the depletion regimen for neutrophils was highly effective in the genital tract, as neutrophils remained depleted throughout secondary infection (Fig. 7A). However, although genital tract neutrophils were effectively depleted at 21 days postinfection (dpi), splenic neutrophils numbers were comparable to those in wild-type mice at that time (Fig. 4B). These data suggest that anti-Ly6G was able to effectively deplete neutrophils in the genital tissues throughout the course of infection but that splenic neutrophils began to repopulate the spleen by 21 days postinfection.

### Delaying neutrophil depletion has no effect on antibody-mediated protection.

**FIG 8:**
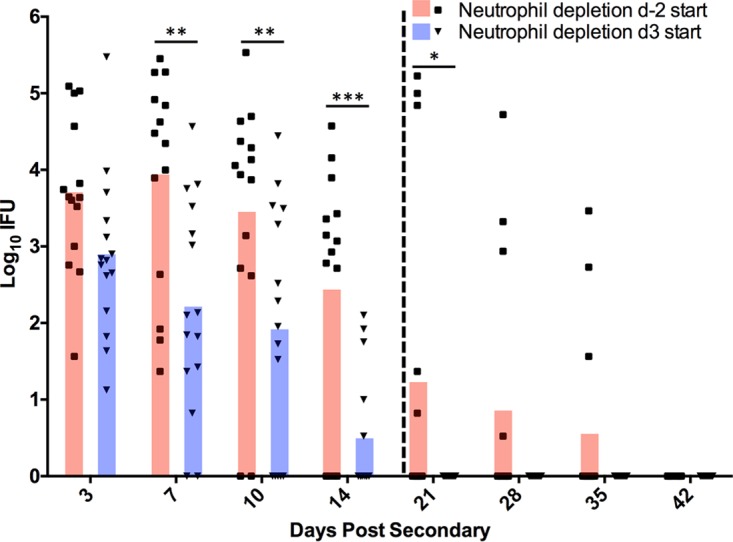
Effect of delayed neutrophil depletion on bacterial clearance during secondary C. muridarum infection. At 66 days after primary infection, C57BL/6 mice depleted of CD4^+^ T cells and neutrophils were rechallenged with C. muridarum. Beginning 6 days prior to rechallenge, anti-CD4 depletion was initiated in all mice. Anti-Ly6G treatment was initiated either 2 days prior to rechallenge (day −2 start) (*n* = 14) or on day 3 (day 3 start) (*n* = 15) following rechallenge. Chlamydial shedding (in IFU) was quantified from cervicovaginal swabs collected throughout secondary infection. The vertical dashed line represents the last dose of anti-CD4 and anti-Ly6G at days 20 and 21, respectively. Data are presented as mean IFU. *, *P* < 0.05; **, *P* < 0.01; ***, *P* < 0.001.

Because of the significant variation in chlamydial burdens (from 10^5^ inclusion-forming units [IFU] to undetectable) seen at later time points (day 10 and onward) in mice depleted of neutrophils and CD4^+^ T cells (Fig. 5), we hypothesized that perhaps effective depletion of neutrophils for an extended period (2 weeks or more) might not be achievable, which would result in premature infection resolution in some mice. Support for this hypothesis came from ineffective splenic depletion of neutrophils at 21 dpi, despite being depleted from genital tract tissue throughout the depletion period (Fig. 7B). To test this hypothesis, we altered the neutrophil depletion regimen. C57BL/6 mice that had resolved primary chlamydial genital infection were depleted of CD4^+^ T cells per our standard depletion regimen. Either neutrophil depletion was accomplished using our standard depletion regimen (depletion was initiated at 2 days prior to infection as for Fig. 5), or depletion was delayed until 3 dpi. If ineffective long-term neutrophil depletion was responsible for infection resolution in our previous experiments, we would expect the delayed depletion regimen to result in an extended, persisting infection for all mice within the treatment group. However, mice receiving the delayed depletion regimen resolved secondary infection significantly more rapidly than control depleted mice (Fig. 8), suggesting that the presence of neutrophils early during rechallenge and/or additional effector cell populations are capable of aiding in antibody-mediated resolution.

### IFN-γ-stimulated neutrophils kill Chlamydia in an antibody-dependent manner *in vitro*.

**FIG 9:**
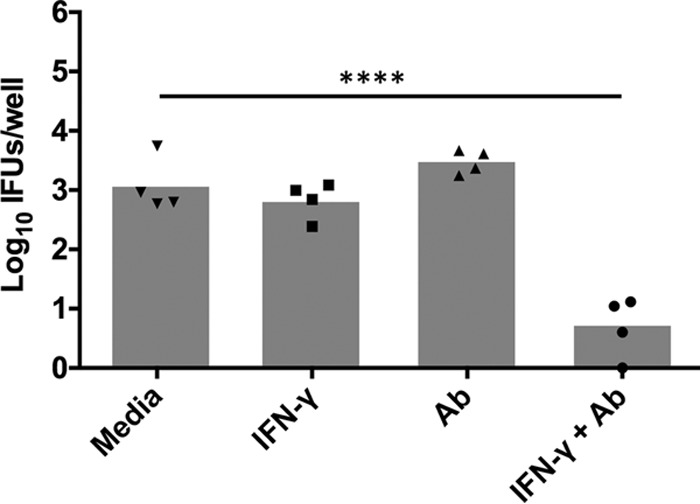
IFN-γ and immune serum are required for antichlamydial neutrophil activity *in vitro*. PPNs were incubated with C. muridarum and either medium, IFN-γ, immune serum (Ab), or IFN-γ and immune serum. PPN lysates were harvested at 43 hpi, and viable chlamydiae (IFU) were enumerated on HeLa 229 cell monolayers. Data represent the mean chlamydial burden (per well) from 4 individual experiments. ****, *P* < 0.0001.

To further investigate the role of neutrophils in antibody-mediated protection, we used an *in vitro* phagocytic killing assay and primary peritoneal neutrophils (PPNs) isolated from C57BL/6 mice. IFN-γ-stimulated or nonstimulated PPNs were incubated with chlamydial elementary bodies (EBs) (with or without immune serum). At 43 h postinfection (hpi), PPN lysates were harvested and viable bacteria were enumerated. IFN-γ-stimulated PPNs, as well as nonstimulated PPNs with immune serum, were incapable of reducing the bacterial burden below that of the nonstimulated control (Fig. 9). However, IFN-γ-stimulated PPNs with immune serum significantly inhibited chlamydiae (approximately 100-fold). At high concentrations of IFN-γ (>1 ng/ml), complete inhibition of chlamydiae was observed regardless of antibody, which is consistent with the well-documented inhibitory activity of higher doses of IFN-γ on Chlamydia (22–25). Interestingly, there was no reduction in bacterial burden when chlamydiae were incubated with antibody and PPNs stimulated with low concentrations of IFN-γ (<0.1 ng/ml). Thus, these data support an IFN-γ-dependent antibody-mediated mechanism of chlamydial killing by neutrophils.

## DISCUSSION

Development of an effective chlamydial vaccine is dependent on defining immune effector mechanisms that display natural protective abilities *in vivo*. Antibody and CD4^+^ T cells have evolved independent mechanisms of chlamydial control, yet their precise mechanisms of protective immunity have not been thoroughly defined (12). In this study, we sought to demonstrate that the protective efficacy of antibody in immunity to genital chlamydia infection relied on antibody/effector cell interactions.

Antibody protects against a variety of pathogens by blocking attachment of the microbe to susceptible host cells (i.e., direct neutralization) (26–28). However, other notable mechanisms of antibody-mediated protection, such as ADCC and opsonization with phagocytic killing, require cooperation between antibody and an effector cell population. We have previously noted that the humoral protection observed following chlamydia genital infection is not due to direct neutralization but is dependent on cooperation between antibody and an effector cell population(s) (9, 13). That conclusion is based upon the findings that passively transferred immune serum fails to protect naive mice from infectious challenge and that antibody-mediated protection is absolutely dependent on CD4^+^ T cell priming of the genital tract tissues. In our current study, we extend our understanding of antichlamydial immunity by demonstrating that protection is mediated in part by IgG and neutrophils.

We initially hypothesized that IgG was likely to play a significant role in protection because it is the dominant class present in antichlamydial immune sera (Fig. 2), and it is capable of mediating a variety of effector functions upon binding to one of its cognate FcRs, including ADCC, and enhancing phagocytosis and microbial killing (29). NK cells are the classical mediators of ADCC, and while our studies do not rule out the possibility of ADCC via other effector populations (14), NK cells appear not to be involved in chlamydial immunity to reinfection in any measurable way. Neutrophils express high levels of the FcRs required to bind antibody/pathogen complexes during infections and mediate pathogen killing via ADCC, phagocytosis and lysosomal degradation, granule release, and a variety of other mechanisms. Neutrophils have traditionally been associated with increased inflammatory pathology during chlamydial infection (30–34), but their role in antibody-mediated immunity had not been assessed. Effective depletion of neutrophils at early time points (Fig. 7) in combination with CD4^+^ T cell depletion resulted in a persisting infection that was significantly higher than in mice depleted of CD4^+^ T cells alone (Fig. 5). Additionally, neutrophil killing of chlamydiae was dependent on the presence of immune serum and IFN-γ stimulation *in vitro* (Fig. 9), consistent with both current and previous studies on *in vivo* antibody-mediated protection (17). From these data, we hypothesize that neutrophils phagocytose and kill chlamydiae following antibody opsonization of the pathogen; however, other mechanisms of neutrophil-mediated killing *in vivo* cannot be ruled out. Thus, neutrophils represent a cell population that is required for optimal antibody-mediated protection against chlamydial reinfection.

Although our data strongly support a role for neutrophils in immunity to genital chlamydia reinfection, there was considerable variation in chlamydial shedding in depleted animals (Fig. 5). That variation might be attributable to other FcR-bearing cell populations, such as macrophages, or to incomplete depletion of neutrophils. Neutrophils are rapidly generated in the bone marrow in response to infection (35, 36), and this response is heightened in the presence of a depleting antibody. Additionally, partially immature neutrophil progenitor cells are released by the bone marrow in an attempt to compensate, likely resulting in a mixed ability of these effectors to kill Chlamydia. This hypothesis was supported by the presence of neutrophils in the spleens of anti-Ly6G-treated mice at 21 dpi (Fig. 7B). In an attempt to ensure effective neutrophil depletion (and possibly delay infection resolution), we delayed the beginning of neutrophil depletion from 2 days prior to infection (day −2) until 3 days after infection (3 dpi), a time when all anti-CD4-treated mice were positively shedding infectious chlamydiae (Fig. 5). However, mice with delayed neutrophil depletion resolved infection more rapidly than those with a normal depletion regimen, indicating that the presence of neutrophils early is adequate to provide protection against reinfection (Fig. 8).

Macrophages are another cell population that fit the criteria for a potential effector cell that functions with antibody to protect against microbial pathogens, including Chlamydia. Macrophages are activated by IFN-γ, express high levels of FcRs, are present in large quantities in the genital tissues during C. muridarum infection, and are capable of killing bacterial pathogens by a variety of different immune mechanisms (14, 16, 17, 37–40). However, our numerous attempts using a variety of methodologies to deplete macrophages in the genital tract during the course of secondary infection have been unsuccessful. Although we suspect that macrophages might also be involved in antibody-mediated chlamydial immunity, we have thus far been unable to effectively evaluate their contribution.

Our studies have clearly demonstrated a key role for IgG and neutrophils in protective immunity to chlamydial genital infection. Absent from our findings, however, is any evidence of a role for IgA in immunity to this mucosal pathogen. Because IgA is known to protect against a variety of mucosal pathogens via both direct and indirect mechanisms (41–43) and because chlamydiae infect, grow, and cause disease at mucosal surfaces, it is surprising that IgA has not been shown to be central to immunity to chlamydial infection. Although no direct evidence supporting a protective role for IgA in immunity to chlamydia genital infection has emerged using the murine model, it cannot be concluded from the available data that IgA is unimportant. Indeed, experimentally addressing the role of IgA *in vivo* is very challenging. For example, we previously showed that IgA-deficient mice resolved primary and secondary chlamydial genital infections similarly to IgA-sufficient mice (44). While those results demonstrate that IgA is not specifically required for immunity, such studies are inconclusive as to the possible contribution of IgA to protection. Similarly, there are caveats when interpreting data from experiments assessing immunity through the passive transfer of serum (9, 11, 13). Although immune serum contains Chlamydia-specific IgA, the IgA is removed by the liver and excreted into the gut upon transfer (45); thus, passively transferred serum is functionally depleted of IgA. Indeed, a few studies do support the notion that IgA could be a factor contributing to immunity (46–49). Higher local levels of IgA have been correlated with both active infection and subsequent infection resolution in humans (47). Although multiple mechanisms of antibody-mediated immunity likely exist, potentially utilizing multiple isotypes to elicit protection, it is clear that further studies are particularly needed to more clearly define the role of IgA in immunity to chlamydia infection.

A key difference between the C. muridarum infection model and human chlamydial infection is the magnitude of cellular infiltration into genital tract tissues following infection. C. muridarum causes marked inflammation characterized by a massive emigration of immune cell populations into the murine genital tract (17, 50). Conversely, the majority the human genital chlamydial infections cause few signs or symptoms of infection (4), suggesting that the local cellular infiltrate is more modest. Therefore, perhaps the infiltrate that follows many human genital infections is inadequate for long-lived natural protection against C. trachomatis, unlike the long-lasting immunity found for the C. muridarum infection model (50, 51). In the mouse, we have shown marked infiltration of the local genital tract tissues with CD4^+^ T cells, which remain long after infection has resolved (50), as well as specific effector populations (neutrophils and perhaps others) that are required for antibody-mediated protection against C. muridarum (17). This robust local immune response is likely key to protective immunity in the murine model. Our data show that IgG is a protective antichlamydial antibody isotype in mice and is protective only in combination with an effector cell population. Although humans mount a significant and long-lasting IgG response (52), perhaps antibody-mediated immunity in humans is more short-lived because the cellular infiltration into the local genital tissues is more transient and less robust. Thus, there may be insufficient numbers of local FcR-bearing effector cells for long-lasting IgG-mediated protection in humans.

In this study, we provide direct evidence detailing components of antibody-mediated protection against genital Chlamydia infection. Importantly, Chlamydia-specific IgG confers protective immunity comparable to that of whole convalescent-phase serum. Additionally, while multiple FcR-bearing populations are capable of interacting with antibody/pathogen complexes, a primary role for neutrophils in antibody-mediated immunity has been characterized, whereas NK cells appeared to be dispensable. Eliciting the appropriate effector responses will be fundamental in the development of an efficacious chlamydial vaccine, highlighting the need to further understand which populations contribute to protective immunity following natural genital infection.

## MATERIALS AND METHODS

### Mice.

Female 6- to 10-week-old wild-type C57BL/6, μMT (antibody-deficient) (B6.129S2-*Igh-6*^*tm1/Cgn*/J^), and beige (C57BL/6J-*Lyst*^*bg-J*/J^) mice were purchased from Jackson Laboratory and housed in the animal facility at the University of Arkansas for Medical Sciences in Little Rock, AR. All institutional guidelines were followed, and protocols were approved by the Institutional Animal Care and Use Committee.

### Chlamydia growth and purification.

C. muridarum (Weiss strain) was propagated in HeLa 229 cells, and infectious elementary bodies (EBs) were harvested and purified via Renografin (Sourceone Healthcare Technologies) discontinuous density gradient centrifugation (53).

### Genital infection and quantitation of chlamydiae.

Prior to primary or secondary infection, mice were treated with medroxyprogesterone acetate (Depo-Provera) (Greenstone LLC) and infected intravaginally with 5 × 10^4^ C. muridarum inclusion-forming units (IFU) (17). Genital infection was assessed at various time points following primary and secondary infections by enumerating IFU (viable bacteria) on HeLa229 cell monolayers (17).

### Immune cell depletions.

CD4^+^ T cells were depleted via intraperitoneal (i.p.) injections of anti-CD4 (clone GK1.5) as previously described (11). Briefly, prior to infectious rechallenge, mice were injected i.p. with 0.5 mg of GK1.5 on days −6, −5, and −4, and then 0.3 mg was administered every 3 days beginning on day −1 and continuing through the indicated times. Following this dosing regimen, CD4^+^ T cells remain depleted (>99%) in the genital tissues and spleen throughout infection and for approximately 7 days following the final dose of anti-CD4 (9).

NK cells were depleted via i.p. injections of anti-NK1.1 (clone PK136). For depletion prior to primary infection, mice were given 0.3 mg of anti-NK1.1 on day −6, day −5, and day −4 and then every 3 days beginning on day −1, continuing through day 38. For depletion prior to secondary rechallenge infection, mice were given 0.3 mg of anti-NK1.1 on day −6, day −5, and day −4 and then every 3 days beginning on day −1, continuing through day 26. Efficient NK cell depletion with this antibody regimen was described previously (54–56).

Neutrophils were depleted via i.p. injections of anti-Ly6G (clone RB6-8C5). For depletion prior to rechallenge, 0.5 mg was administered i.p. on day −2 and day 0, and then 0.3-mg injections were administered every 3 days beginning on day 2 and continuing through day 21. In one experiment, we used a delayed neutrophil depletion scheme. For that depletion, 0.5 mg was administered i.p. on day 3 and day 5, and then 0.3-mg injections were given every 3 days beginning on day 8 and continuing through day 21. The efficacy of neutrophil depletion is shown in Fig. 7.

### Immune serum preparations and passive transfer.

Convalescent (immune) serum, collected from mice having resolved a primary C. muridarum genital infection, was used to test the protective efficacy of various immunoglobulin fractions. Serum was first fractionated using protein A-Sepharose 4B affinity chromatography. The unbound fraction (IgG-depleted flowthrough) was collected and saved. The bound IgG was eluted using 0.1 M glycine (pH 2.8) and collected directly into tubes containing 2 M Tris, pH 8.0. Antibody-containing fractions were extensively dialyzed against 10 mM phosphate-buffered saline (PBS), and the volume of the purified IgG was adjusted to the same volume as the starting serum. Fab fragments were generated from the purified IgG fraction using an ImmunoPure Fab preparation kit (57) following the manufacturer's instructions. The homogeneity of the Fab preparation was confirmed by SDS-PAGE. To test protective efficacy, 0.5 ml of serum or fraction thereof (described above) was passively transferred i.p. to recipient CD4-depleted antibody-deficient mice beginning 1 day prior to infection (day −1) and then on days 0, 3, 6, 9, 12, and 15 postinfection.

### Chlamydial ELISA.

Chlamydia-specific serum antibody was measured by ELISAs using formalin-fixed, density gradient-purified C. muridarum elementary bodies (EBs) as the antigen as previously described (10).

### Flow cytometry.

Whole murine genital tracts and spleens were isolated at the indicated time points as previously described in detail (17). Genital tract cells and splenocytes were stained with the following antibodies in fluorescence-activated cell sorting (FACS) buffer: anti-Ly6G (Gr-1)-eFluor 450 (RB6-8C5; eBioscience) and viability dye-eFluor 780 (eBioscience). The gating strategy was determined using fluorescence minus one (FMO) controls (58).

### PPN isolation.

Primary peritoneal neutrophils (PPNs) were elicited in C57BL/6 mice via i.p. injection of 1 ml of 90 mg/ml casein (59) at 24 h prior to harvest, followed by one additional 1-ml injection at 3 h prior to PPN collection. PPNs were harvested by irrigating the peritoneal cavities of euthanized mice with 5 to 10 ml sterile PBS. Cells were washed three times in sterile PBS, resuspended in sterile PBS, and counted using an Auto 100 Cellometer (Nexcelom Bioscience).

### *In vitro* phagocytic killing assay.

C. muridarum EBs at 1 × 10^6^/ml were incubated alone or with immune serum (final dilution of 1:50) in 0.5 ml Dulbecco's modified Eagle medium–nutrient mixture F-12 (DMEM-F12) (Life Technologies) rotating for 1 h at 37°C. Separately, PPNs were incubated alone or with 0.5 ng/ml recombinant mouse IFN-γ (BD Biosciences) in DMEM-F12 rotating for 1 h at 37°C. EB-serum and PPN–IFN-γ samples were combined at 1:5, respectively (representing a multiplicity of infection of 0.1) and mixed rotating for 1 h at 37°C. Samples were then plated in 0.5 ml at 1 × 10^6^ PPNs/well in 48-well cell culture plates and rested for 30 min at 37°C. Monolayers were washed three times with Hanks' balanced salt solution (HBSS) to remove nonadherent cells. Fresh DMEM–F12-10 (DMEM-F12 plus 10% fetal bovine serum), was added and infection was allowed to proceed at 37°C. At 43 hpi, PPN lysates were collected via scraping in a 0.5-ml mixture of 250 mM sucrose, 10 mM sodium phosphate, and 5 mM l-glutamic acid (pH 7.2) (SPG). Lysate samples were homogenized by sequential passage through 27- and 31-gauge needles (BD Biosciences). Lysates were stored at −80°C until IFU enumeration.

### Statistical analysis.

IFU data were analyzed using two-way analysis of variance (ANOVA) with the Bonferroni posttest. One-way ANOVA with the Bonferroni posttest was used for the analysis of antibody titer and *in vitro* IFU data. Statistics were calculated using Prism 6.0f.

## References

[1] World Health Organization. 2012 Global incidence and prevalence of selected curable sexually transmitted infections—2008. World Health Organization, Geneva, Switzerland.

[2] Newman L, Rowley J, Vander Hoorn S, Wijesooriya NS, Unemo M, Low N, Stevens G, Gottlieb S, Kiarie J, Temmerman M 2015 Global estimates of the prevalence and incidence of four curable sexually transmitted infections in 2012 based on systematic review and global reporting. PLoS One 10:e0143304. doi:10.1371/journal.pone.0143304. 26646541 PMC4672879

[3] Owusu-Edusei K Jr, Chesson HW, Gift TL, Tao G, Mahajan R, Ocfemia MC, Kent CK 2013 The estimated direct medical cost of selected sexually transmitted infections in the United States, 2008. Sex Transm Dis 40:197–201. doi:10.1097/OLQ.0b013e318285c6d2. 23403600

[4] Farley TA, Cohen DA, Elkins W 2003 Asymptomatic sexually transmitted diseases: the case for screening. Prev Med 36:502–509. doi:10.1016/S0091-7435(02)00058-0. 12649059

[5] Haggerty CL, Panum I, Uldum SA, Bass DC, Olsen J, Darville T, Eastman JM, Simhan HN, Roberts JM, Ness RB 2013 *Chlamydia trachomatis* infection may increase the risk of preeclampsia. Pregnancy Hypertens 3:28–33. doi:10.1016/j.preghy.2012.09.002. 26105737

[6] Haggerty CL, Gottlieb SL, Taylor BD, Low N, Xu F, Ness RB 2010 Risk of sequelae after *Chlamydia trachomatis* genital infection in women. J Infect Dis 201(Suppl 2):S134–S155. doi:10.1086/652395. 20470050

[7] Cates W Jr, Wasserheit JN 1991 Genital chlamydial infections: epidemiology and reproductive sequelae. Am J Obstet Gynecol 164:1771–1781. doi:10.1016/0002-9378(91)90559-A. 2039031

[8] Su H, Caldwell HD 1995 CD4+ T cells play a significant role in adoptive immunity to *Chlamydia trachomatis* infection of the mouse genital tract. Infect Immun 63:3302–3308. 7642259 10.1128/iai.63.9.3302-3308.1995 PMC173455

[9] Morrison SG, Su H, Caldwell HD, Morrison RP 2000 Immunity to murine *Chlamydia trachomatis* genital tract reinfection involves B cells and CD4(+) T cells but not CD8(+) T cells. Infect Immun 68:6979–6987. doi:10.1128/IAI.68.12.6979-6987.2000. 11083822 PMC97807

[10] Morrison RP, Feilzer K, Tumas DB 1995 Gene knockout mice establish a primary protective role for major histocompatibility complex class II-restricted responses in *Chlamydia trachomatis* genital tract infection. Infect Immun 63:4661–4668. 7591120 10.1128/iai.63.12.4661-4668.1995 PMC173669

[11] Morrison SG, Morrison RP 2001 Resolution of secondary *Chlamydia trachomatis* genital tract infection in immune mice with depletion of both CD4+ and CD8+ T cells. Infect Immun 69:2643–2649. doi:10.1128/IAI.69.4.2643-2649.2001. 11254630 PMC98202

[12] Morrison RP, Caldwell HD 2002 Immunity to murine chlamydial genital infection. Infect Immun 70:2741–2751. doi:10.1128/IAI.70.6.2741-2751.2002. 12010958 PMC128027

[13] Morrison SG, Morrison RP 2005 A predominant role for antibody in acquired immunity to chlamydial genital tract reinfection. J Immunol 175:7536–7542. doi:10.4049/jimmunol.175.11.7536. 16301662 PMC3514507

[14] Zighelboim J, Bonavida B, Fahey JL 1973 Evidence for several cell populations active in antibody dependent cellular cytotoxicity. J Immunol 111:1737–1742. 4750865

[15] Tseng CT, Rank RG 1998 Role of NK cells in early host response to chlamydial genital infection. Infect Immun 66:5867–5875. 9826367 10.1128/iai.66.12.5867-5875.1998 PMC108743

[16] Moore T, Ananaba GA, Bolier J, Bowers S, Belay T, Eko FO, Igietseme JU 2002 Fc receptor regulation of protective immunity against *Chlamydia trachomatis*. Immunology 105:213–221. doi:10.1046/j.0019-2805.2001.01354.x. 11872097 PMC1782645

[17] Naglak EK, Morrison SG, Morrison RP 6 9 2016 IFNγ is required for optimal antibody-mediated immunity against genital chlamydia infection. Infect Immun doi:10.1128/IAI.00749-16. PMC5067755 27600502

[18] Schiff DE, Rae J, Martin TR, Davis BH, Curnutte JT 1997 Increased phagocyte Fc gammaRI expression and improved Fc gamma-receptor-mediated phagocytosis after *in vivo* recombinant human interferon-gamma treatment of normal human subjects. Blood 90:3187–3194. 9376602

[19] Schroder K, Hertzog PJ, Ravasi T, Hume DA 2004 Interferon-gamma: an overview of signals, mechanisms and functions. J Leukoc Biol 75:163–189. doi:10.1189/jlb.0603252. 14525967

[20] Daley JM, Thomay AA, Connolly MD, Reichner JS, Albina JE 2008 Use of Ly6G-specific monoclonal antibody to deplete neutrophils in mice. J Leukoc Biol 83:64–70. doi:10.1189/jlb.0407247. 17884993

[21] Byrne GI, Stephens RS, Ada G, Caldwell HD, Su H, Morrison RP, Van der Pol B, Bavoil P, Bobo L, Everson S, Ho Y, Hsia RC, Kennedy K, Kuo C-C, Montgomery PC, Peterson E, Swanson A, Whitaker C, Whittum-Hudson J, Yang CL, Zhang Y-X, Zhong GM 1993 Workshop on *in vitro* neutralization of *Chlamydia trachomatis*: summary of proceedings. J Infect Dis 168:415–420. doi:10.1093/infdis/168.2.415. 8335979

[22] Hanna L, Merigan TC, Jawetz E 1966 Inhibition of TRIC agents by virus-induced interferon. Proc Soc Exp Biol Med 122:417–421. doi:10.3181/00379727-122-31150. 5980548

[23] Kazar J, Gillmore JD, Gordon FB 1971 Effect of interferon and interferon inducers on infections with a nonviral intracellular microorganism, *Chlamydia trachomatis*. Infect Immun 3:825–832. 16558062 10.1128/iai.3.6.825-832.1971 PMC416245

[24] Beatty WL, Byrne GI, Morrison RP 1993 Morphologic and antigenic characterization of interferon gamma-mediated persistent *Chlamydia trachomatis* infection in vitro. Proc Natl Acad Sci U S A 90:3998–4002. doi:10.1073/pnas.90.9.3998. 8387206 PMC46433

[25] Nelson DE, Virok DP, Wood H, Roshick C, Johnson RM, Whitmire WM, Crane DD, Steele-Mortimer O, Kari L, McClarty G, Caldwell HD 2005 Chlamydial IFN-gamma immune evasion is linked to host infection tropism. Proc Natl Acad Sci U S A 102:10658–10663. doi:10.1073/pnas.0504198102. 16020528 PMC1180788

[26] Colonno RJ, Callahan PL, Long WJ 1986 Isolation of a monoclonal antibody that blocks attachment of the major group of human rhinoviruses. J Virol 57:7–12. 3001366 10.1128/jvi.57.1.7-12.1986 PMC252692

[27] Chanock RM, Crowe JE Jr, Murphy BR, Burton DR 1993 Human monoclonal antibody Fab fragments cloned from combinatorial libraries: potential usefulness in prevention and/or treatment of major human viral diseases. Infect Agents Dis 2:118–131. 8173784

[28] Kato H, Kato R, Fujihashi K, McGhee JR 2001 Role of mucosal antibodies in viral infections. Curr Top Microbiol Immunol 260:201–228. 11443875 10.1007/978-3-662-05783-4_11

[29] Schroeder HW Jr, Cavacini L 2010 Structure and function of immunoglobulins. J Allergy Clin Immunol 125:S41–S52. doi:10.1016/j.jaci.2009.09.046. 20176268 PMC3670108

[30] Andrew DW, Cochrane M, Schripsema JH, Ramsey KH, Dando SJ, O'Meara CP, Timms P, Beagley KW 2013 The duration of *Chlamydia muridarum* genital tract infection and associated chronic pathological changes are reduced in IL-17 knockout mice but protection is not increased further by immunization. PLoS One 8:e76664. doi:10.1371/journal.pone.0076664. 24073293 PMC3779189

[31] Riley MM, Zurenski MA, Frazer LC, O'Connell CM, Andrews CW Jr, Mintus M, Darville T 2012 The recall response induced by genital challenge with C*hlamydia muridarum* protects the oviduct from pathology but not from reinfection. Infect Immun 80:2194–2203. doi:10.1128/IAI.00169-12. 22431649 PMC3370586

[32] Frazer LC, O'Connell CM, Andrews CW Jr, Zurenski MA, Darville T 2011 Enhanced neutrophil longevity and recruitment contribute to the severity of oviduct pathology during *Chlamydia muridarum* infection. Infect Immun 79:4029–4041. doi:10.1128/IAI.05535-11. 21825059 PMC3187238

[33] Scurlock AM, Frazer LC, Andrews CW Jr, O'Connell CM, Foote IP, Bailey SL, Chandra-Kuntal K, Kolls JK, Darville T 2011 Interleukin-17 contributes to generation of Th1 immunity and neutrophil recruitment during *Chlamydia muridarum* genital tract infection but is not required for macrophage influx or normal resolution of infection. Infect Immun 79:1349–1362. doi:10.1128/IAI.00984-10. 21149587 PMC3067500

[34] Zhang H, Zhou Z, Chen J, Wu G, Yang Z, Zhou Z, Baseman J, Zhang J, Reddick RL, Zhong G 2014 Lack of long-lasting hydrosalpinx in A/J mice correlates with rapid but transient chlamydial ascension and neutrophil recruitment in the oviduct following intravaginal inoculation with *Chlamydia muridarum*. Infect Immun 82:2688–2696. doi:10.1128/IAI.00055-14. 24711570 PMC4097620

[35] Furze RC, Rankin SM 2008 Neutrophil mobilization and clearance in the bone marrow. Immunology 125:281–288. doi:10.1111/j.1365-2567.2008.02950.x. 19128361 PMC2669132

[36] Zhao E, Xu H, Wang L, Kryczek I, Wu K, Hu Y, Wang G, Zou W 2012 Bone marrow and the control of immunity. Cell Mol Immunol 9:11–19. doi:10.1038/cmi.2011.47. 22020068 PMC3251706

[37] MacGowan AP, Peterson PK, Keane W, Quie PG 1983 Human peritoneal macrophage phagocytic, killing, and chemiluminescent responses to opsonized *Listeria monocytogenes*. Infect Immun 40:440–443. 6403471 10.1128/iai.40.1.440-443.1983 PMC264869

[38] Leu RW, Rummage JA, Horn MJ 1989 Characterization of murine macrophage Fc receptor-dependent phagocytosis and antibody-dependent cellular cytotoxicity during in vitro culture with interferons-gamma, alpha/beta and/or fetal bovine serum. Immunobiology 178:340–350. doi:10.1016/S0171-2985(89)80057-9. 2469639

[39] Jorens PG, Matthys KE, Bult H 1995 Modulation of nitric oxide synthase activity in macrophages. Mediators Inflamm 4:75–89. doi:10.1155/S0962935195000135. 18475620 PMC2365621

[40] Brieland JK, Heath LA, Huffnagle GB, Remick DG, McClain MS, Hurley MC, Kunkel RK, Fantone JC, Engleberg C 1996 Humoral immunity and regulation of intrapulmonary growth of *Legionella pneumophila* in the immunocompetent host. J Immunol 157:5002–5008. 8943407

[41] Blutt SE, Miller AD, Salmon SL, Metzger DW, Conner ME 2012 IgA is important for clearance and critical for protection from rotavirus infection. Mucosal Immunol 5:712–719. doi:10.1038/mi.2012.51. 22739233 PMC3461240

[42] Belyakov IM, Ahlers JD 2009 What role does the route of immunization play in the generation of protective immunity against mucosal pathogens? J Immunol 183:6883–6892. doi:10.4049/jimmunol.0901466. 19923474

[43] Macpherson AJ, McCoy KD, Johansen FE, Brandtzaeg P 2008 The immune geography of IgA induction and function. Mucosal Immunol 1:11–22. doi:10.1038/mi.2007.6. 19079156

[44] Morrison SG, Morrison RP 2005 The protective effect of antibody in immunity to murine chlamydial genital tract reinfection is independent of immunoglobulin A. Infect Immun 73:6183–6186. doi:10.1128/IAI.73.9.6183-6186.2005. 16113345 PMC1231110

[45] Russell MW, Brown TA, Mestecky J 1982 Preferential transport of IgA and IgA-immune complexes to bile compared with other external secretions. Mol Immunol 19:677–682. doi:10.1016/0161-5890(82)90369-8. 7110141

[46] Cotter TW, Meng Q, Shen ZL, Zhang YX, Su H, Caldwell HD 1995 Protective efficacy of major outer membrane protein-specific immunoglobulin A (IgA) and IgG monoclonal antibodies in a murine model of *Chlamydia trachomatis* genital tract infection. Infect Immun 63:4704–4714. 7591126 10.1128/iai.63.12.4704-4714.1995 PMC173675

[47] Brunham RC, Kuo CC, Cles L, Holmes KK 1983 Correlation of host immune response with quantitative recovery of *Chlamydia trachomatis* from the human endocervix. Infect Immun 39:1491–1494. 6840846 10.1128/iai.39.3.1491-1494.1983 PMC348123

[48] Fresse AS, Sueur JM, Hamdad F 2010 Diagnosis and follow-up of genital chlamydial infection by direct methods and by detection of serum IgG, IgA and secretory IgA. Indian J Med Microbiol 28:326–331. doi:10.4103/0255-0857.71823. 20966563

[49] Armitage CW, O'Meara CP, Harvie MC, Timms P, Wijburg OL, Beagley KW 2014 Evaluation of intra- and extra-epithelial secretory IgA in chlamydial infections. Immunology 143:520–530. doi:10.1111/imm.12317. 24827556 PMC4253500

[50] Morrison SG, Morrison RP 2000 *In situ* analysis of the evolution of the primary immune response in murine *Chlamydia trachomatis* genital tract infection. Infect Immun 68:2870–2879. doi:10.1128/IAI.68.5.2870-2879.2000. 10768984 PMC97499

[51] Ramsey KH, Newhall WJt Rank RG 1989 Humoral immune response to chlamydial genital infection of mice with the agent of mouse pneumonitis. Infect Immun 57:2441–2446. 2744854 10.1128/iai.57.8.2441-2446.1989 PMC313467

[52] Geisler WM, Morrison SG, Doemland ML, Iqbal SM, Su J, Mancevski A, Hook EW III, Morrison RP 2012 Immunoglobulin-specific responses to *Chlamydia* elementary bodies in individuals with and at risk for genital chlamydial infection. J Infect Dis 206:1836–1843. doi:10.1093/infdis/jis621. 23045619 PMC3502381

[53] Caldwell HD, Kromhout J, Schachter J 1981 Purification and partial characterization of the major outer membrane protein of *Chlamydia trachomatis*. Infect Immun 31:1161–1176. 7228399 10.1128/iai.31.3.1161-1176.1981 PMC351439

[54] Wang M, Ellison CA, Gartner JG, HayGlass KT 1998 Natural killer cell depletion fails to influence initial CD4 T cell commitment *in vivo* in exogenous antigen-stimulated cytokine and antibody responses. J Immunol 160:1098–1105. 9570522

[55] Harshan KV, Gangadharam PR 1991 *In vivo* depletion of natural killer cell activity leads to enhanced multiplication of Mycobacterium avium complex in mice. Infect Immun 59:2818–2821. 1855997 10.1128/iai.59.8.2818-2821.1991 PMC258091

[56] Weidanz WP, LaFleur G, Brown A, Burns JM Jr, Gramaglia I, van der Heyde HC 2010 Gammadelta T cells but not NK cells are essential for cell-mediated immunity against *Plasmodium chabaudi* malaria. Infect Immun 78:4331–4340. doi:10.1128/IAI.00539-10. 20660608 PMC2950347

[57] Spahn J, Pierce RH, Crispe IN 2011 Ineffective CD8(+) T-cell immunity to adeno-associated virus can result in prolonged liver injury and fibrogenesis. Am J Pathol 179:2370–2381. doi:10.1016/j.ajpath.2011.08.004. 21925469 PMC3204094

[58] Roederer M 2001 Spectral compensation for flow cytometry: visualization artifacts, limitations, and caveats. Cytometry 45:194–205. doi:10.1002/1097-0320(20011101)45:3<194::AID-CYTO1163>3.0.CO;2-C. 11746088

[59] Swamydas M, Luo Y, Dorf ME, Lionakis MS 2015 Isolation of mouse neutrophils. Curr Protoc Immunol 110:3.20.1–3.20.15. doi:10.1002/0471142735.im0320s110. PMC4574512 26237011

